# Peripheral blood T-cell modulation by omalizumab in chronic urticaria patients

**DOI:** 10.3389/fimmu.2024.1413233

**Published:** 2024-08-20

**Authors:** Cristina López, Nathalie Depreux, Isabel Bielsa, Albert Roger, Bibiana Quirant-Sanchez, Maria Basagaña, Yanina Jurgens, Clara Padró, Sira Miquel, Eva Martinez-Caceres, Aina Teniente-Serra

**Affiliations:** ^1^ Immunology Division, Laboratori Clinic Metropolitana Nord (LCMN), Germans Trias i Pujol University Hospital and Research Institute (IGTP), Badalona, Spain; ^2^ Department of Cell Biology, Physiology and Immunology, Universitat Autònoma de Barcelona, Badalona, Spain; ^3^ Allergy Section, Germans Trias i Pujol University Hospital, Badalona, Spain; ^4^ Department of Dermatology, Germans Trias i Pujol University Hospital, Badalona, Spain

**Keywords:** chronic urticaria, autoimmune chronic urticaria, immune profile, omalizumab, immune system

## Abstract

**Background:**

Chronic spontaneous urticaria (CSU) is a highly prevalent and difficult to manage cutaneous disease characterized by the presence of recurrent urticaria, angioedema, or both, for a period of 6 weeks or longer. One of the biological treatments used for patients with CSU with an autoimmune background and bad control of the disease is omalizumab, an anti-IgE monoclonal antibody. The understanding of the mechanism of action of this biological drug in CSU along with the identification of potential biomarkers of clinical response can be helpful in the personalized management of the disease.

**Objective:**

The purpose of this study was to analyze the effect of omalizumab on peripheral blood lymphocyte subpopulations in patients with CSU in order to identify potential biomarkers of treatment response.

**Methods:**

We analyzed 71 patients with CSU [33 under omalizumab and 38 under non-immunomodulatory drugs (treated with antihistamines; NID)] and 50 healthy controls. An exhaustive immunophenotyping of whole blood T-cell subpopulations, including naïve, central memory, effector memory, effector cells, Th1, Th2, and Th17 was performed by multiparametric flow cytometry. Moreover, in CSU patients, we analyzed markers of inflammation (ESR, DD, CRP), atopy (prick test, IgE quantification), and autoimmunity (anti-thyroid antibodies and indirect basophil activation test).To evaluate the clinical activity, the Urticaria Activity Score 7 (UAS 7) test was used.

**Results:**

In patients with CSU under treatment with omalizumab, there was a significant decrease in the percentage of naïve and an increase in the percentage of central memory CD4 T cells as well as a decrease in the percentage of naïve and increase in the percentage of effector CD8 T-cell subsets. Moreover, patients under treatment with omalizumab had higher percentages of Th1 and Th2 cells than patients under treatment with NID.

**Conclusion:**

The immune monitoring of T-cell subpopulations in patients with CSU starting omalizumab, may be a useful strategy to analyze treatment response in the clinical practice.

## 1 Introduction

Chronic spontaneous urticaria (CSU) is defined as the appearance of hive-like lesions that may be accompanied by angioedema and last for 6 or more weeks (1). The disorder is highly prevalent and has an important effect on the patients’ quality of life (2, 3). Unfortunately, little is known about the pathophysiology of the disease. It has been postulated that various hypersensitivity mechanisms could be involved in its pathogenesis. There are various hypersensitive reactions regarding immunological urticaria including type I (mediated by IgE that will lead to acute urticaria symptoms), type II [involved IgG leading to chronic autoimmune urticaria (CAU)], and type IV (mediated by T lymphocytes, potentially involved in CSU). The most recognized mechanisms involved in this pathology are those mediated by IgG autoantibodies (3).

In recent years, a new phenotype has been described, known as CAU, whose mechanism is not fully established. It has been widely accepted the involvement of an autoimmune background supported by the presence of IgG autoantibodies anti-IgE and IgG anti-FcRI and IgE autoantibodies against in sera from patients with CAU (4, 5). These autoantibodies, present in 45%–55% of CAU patients, can activate the classical complement pathway through the formation of immune complexes with IgG and free IgE (4, 5). Additionally, as observed in other autoimmune diseases, the emergence of these IgG autoantibodies is correlated with the presence of additional autoantibodies, primarily antithyroid antibodies (anti-TPO and anti-thyroglobulin), which may trigger autoimmune thyroiditis (6, 7). All these elements underscore the pivotal role of the immune system in the pathophysiology of CSU.

Previous studies analyzing the immunophenotype of CSU patients showed an increase of B cells, controlled mainly by IL-21 and IL-15 cytokines (8). In relation to T lymphocytes, a mixed Th1 and Th2 profile was observed, characterized by the secretion of cytokines such as IL-6, TNF-α, and IFN-γ (8, 9). Furthermore, a reduction in the number of Th17 and regulatory T cells has also been described (9). Regarding activation markers, it has been demonstrated that, in CSU patients, T-cell activation correlates with mast cell degranulation (5, 10). Furthermore, skin biopsies from CAU patients revealed a predominant infiltration of CD4^+^ T cells primarily comprising activated CD4 ^+^ T cells (expressing HLA-DR) (9, 11, 12).

Moreover, an inflammatory background in CU patients has been postulated (13). Several studies correlated the severity of the disease with an increase or positivity of biomarkers of inflammation as D-dimer (DD), C-reactive protein (CRP), and erythrocyte sedimentation rate (ESR) (13–15).

The severity, prognosis, and management of CSU vary among patients, requiring a personalized therapeutic approach. European guidelines recommend the use of antihistamines to manage the disease (1). In cases where this approach proves insufficient, omalizumab treatment may be beneficial (1). Omalizumab is a monoclonal antibody that binds free IgE in humans, preventing its interaction with receptors on mast cells and basophils, thus inhibiting degranulation and the release of products like histamine. Although there are many studies regarding the mechanism of action of omalizumab in CSU, the research on its effect on T-cell subpopulations (specifically in those involved in the autoimmune response, as Th1 and Th2) remains limited (16, 17).

The aim of the present study was to analyze using flow cytometry T lymphocyte subsets in peripheral blood of patients with CSU, to better understand the effect of omalizumab. This will allow us to identify potential biomarkers that would be useful to a more personalized management of the disease.

## 2 Materials and methods

### 2.1 Patients

We carried out a prospective study of 71 patients over the age of 18 years with CSU recruited between 2014 and 2021 at the Departments of Allergology and Dermatology Germans Trias i Pujol Hospital, Barcelona. Within this group, 33 patients were under omalizumab treatment (eight males and 25 females) and 38 (15 males and 23 females) under non-immunomodulatory drugs (treated with antihistamines; NID). Patients who initiated omalizumab treatment after inadequate response to four times the standard dose of antihistamines under medical supervision, following the European guidelines on chronic urticaria (1), were included. The dosing and administration of omalizumab followed the algorithm for CSU treatment with omalizumab established by the urticaria network from Catalonia and Balearic Islands (“Xarxa d’Urticaria Catalana i Balear”) (18).

Patients under 18 years, with another allergic disease, in treatment with another biological drug or under an immunosuppressive state were excluded from the study.

Blood samples were analyzed before and after 6 months of omalizumab treatment, with informed consent obtained from all patients. Data collection was anonymized and conducted in compliance with medical research codes, following approval from Ethics Committee of our hospital (PI-20-034). From the 33 patients under omalizumab treatment, some clinical parameters were taken: associated and type of autoimmune diseases (thyroiditis, if present), associated angioedema (clinical diagnosis), score on the Urticaria Activity Score 7 (UAS 7) scale (for severity of the urticaria), and associated atopy (prick test). All subjects also underwent skin tests to inhalant allergens. Questionnaires assessing CSU control were given before and 6 months post-treatment.

**Figure 1:**
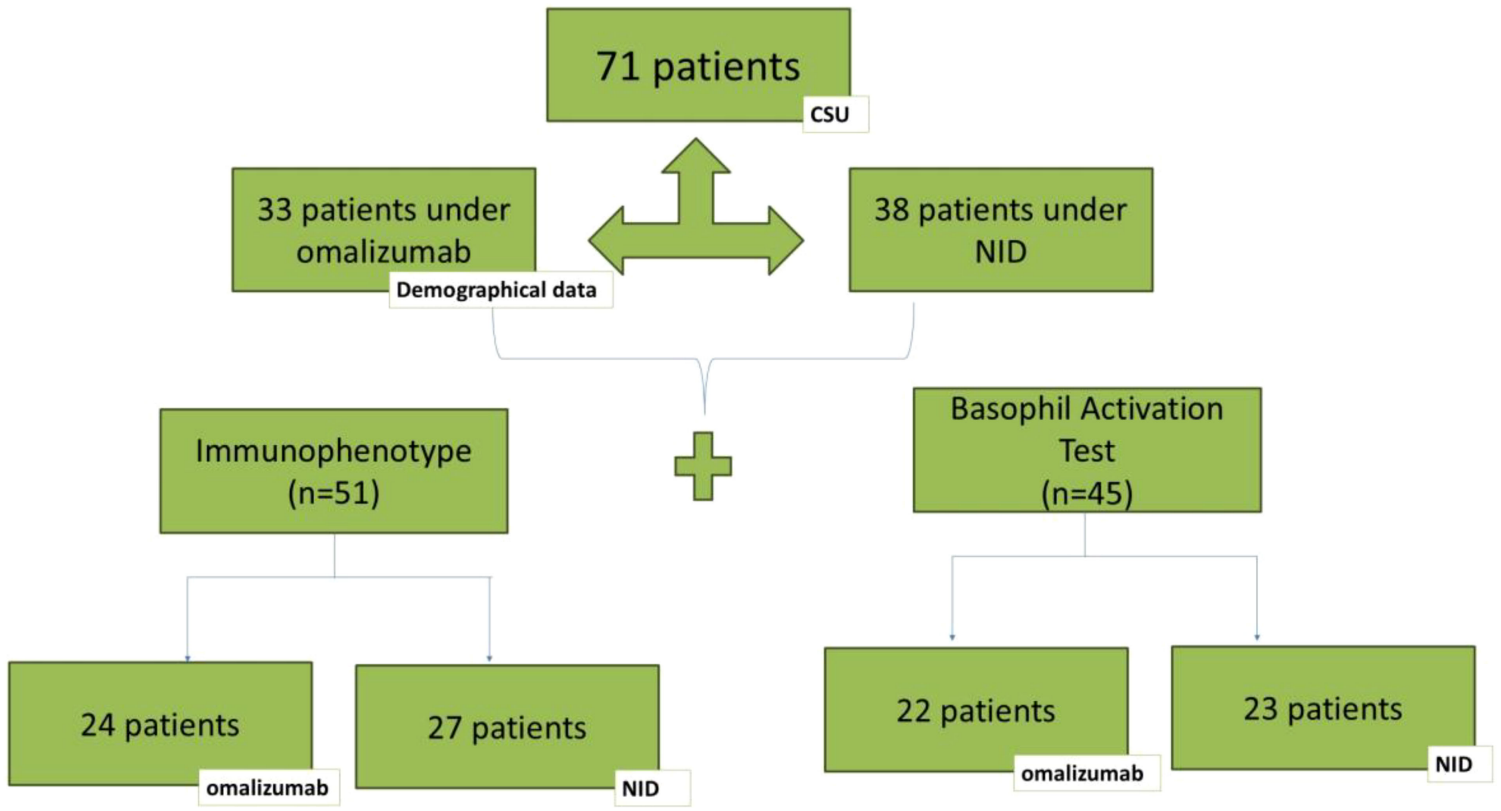
Study design of the patients included in the study. Description of the patients enrolled in the study and the characterization performed in each group.

Regarding the immunological study, indirect Basophil Activation Test (BAT) was conducted on 45 CSU patients (22 receiving omalizumab treatment and 23 with NID). Additionally, for the immunophenotype study, 51 patients of 71 were analyzed by flow cytometry, 27 under NID, and 24 under omalizumab treatment. A control group consisted of fifty healthy donors (HDs) from the Blood and Tissue Bank of Catalonia (BST) were included (28 females, 22 males between 21 and 36 years) (**Figure 1**).

An additional analysis was performed in a subgroup of 38 CSU patients (14 baseline patients and 24 post-treatment) to study lymphocyte subpopulations before and after treatment with omalizumab.

### 2.2 Methods

#### 2.2.1 Skin tests (prick tests)

Prick testing was performed for the following allergens: dust mites (*Dermatophagoides pteronyssinus*, *Dermatophagoides farinae*, *Acarus siro*, *Lepidoglyphus destructor*), fungi (*Alternaria*, *Cladosporium*, *Aspergillus spp*, *Penicillium* spp.), epithelial (dog and cat), tree pollen (*Cupressus sempervivens*, *Platanus acerifolia*, *Pinus radiata*, *Quercus ilex*, *Olea europea*), weeds (*Parietaria judaica*, *Artemisia vulgaris*, *Chenopodium album*, mixture of weeds pollens) and grasses (*Cynodon dactyilis* and *Lollium perennial*). When mixture of weeds pollen tests was positive, a further breakdown was performed using *Plantago ovata*, *Salsola kali* and *Mercurialis annua* (all of them from Leti Laboratories SA).

### 2.3 Biological samples

Blood samples were obtained through venipuncture. For the analysis of the lymphocyte subpopulations, samples were collected in an EDTA tube and were stored at room temperature and analyzed within 4h post-extraction.

ESR and DD were analyzed in plasma using DxH 900 (Beckman Coulter, Indianapolis, USA) and CRP in serum samples using AU-5800 (Beckman Coulter). Furthermore, serum parameters such as total IgE, specific IgE against hidden allergens as Anisakis, Tri a19 and Pru p3 were analyzed using immunoassay (ImmunoCAP^®^, ThermoFisher-Phadia, Massachussetts, USA). Antithyroid autoantibodies were analyzed from sera using chemiluminescence (Abbott Diagnostics, Illinois, USA).

### 2.4 Lymphocyte subpopulations by flow cytometry

A phenotypical characterization of the T lymphocyte populations in whole peripheral blood was carried out. We used the standardized panels included in the European ENTIRE HIP-C protocol (19–21) (**Table 1**).

**Table 1:** Lymphocyte characterization.

Lymphocyte subpopulation	Phenotype
CD8 T-cell subsets	
**CD8^+^ T cell**	CD3^+^CD8^+^
**CD8^+^ naïve T cell**	CD3^+^CD8^+^CCR7^+^CD45RA^+^
**CD8^+^ Central Memory T cell (CM)**	CD3^+^CD8^+^CCR7^+^CD45RA-
**CD8^+^ Effector Memory T cell (EM)**	CD3^+^CD8^+^CCR7^-^CD45RA-
**CD8^+^ Terminally differentiated T cell (TEMRA)**	CD3^+^CD8^+^CCR7^-^CD45RA^+^
**CD8^+^ activated T cell**	CD3^+^CD8^+^HLA-DR^+^CD38^+^/CD3^+^CD8^+^HLA-DR^+^CD38-
CD4 T-cell subsets	
**CD4^+^ T cell**	CD3^+^CD4^+^
**CD4^+^ naïve T cell**	CD3^+^CD4^+^CCR7^+^CD45RA^+^
**CD4^+^ Central Memory T cell (CM)** **Th1 Central Memory** ** Th2 Central Memory** ** Th17 Central Memory**	CD3^+^CD4^+^CCR7^+^CD45RA- CD3^+^CD4^+^CCR7^+^CD45RA-CXCR3^+^CCR6^-^ CD3^+^CD4^+^CCR7^+^CD45RA-CXCR3^-^CCR6- CD3^+^CD4^+^CCR7^+^CD45RA-CXCR3^-^CCR6^+^
**CD4^+^ Effector Memory T cell (EM)** **Th1 Effector Memory** ** Th2 Effector Memory** ** Th17 Effector Memory**	CD3^+^CD4^+^CCR7^-^CD45RA- CD3^+^CD4^+^CCR7^-^CD45RA-CXCR3^+^CCR6^-^ CD3^+^CD4^+^CCR7^-^CD45RA-CXCR3^-^CCR6- CD3^+^CD4^+^CCR7^-^CD45RA-CXCR3^-^CCR6^+^
**CD4^+^ Terminally differentiated T cell (TEMRA)**	CD3^+^CD4^+^CCR7^-^CD45RA^+^
**CD4^+^ activated T cell**	CD3^+^CD4^+^HLA-DR^+^CD38^+^/CD3^+^CD4^+^HLA-DR^+^CD38-

One hundred microliter of whole blood were stained for the surface markers listed in **Table 2** (the majority from BD Biosciences, San José, CA, USA, except for CXCR3, CD45, and CCR6 from BioLegend, San Diego, CA). We followed the protocol of the ENTIRE HIP-C project (21).

**Table 2:** Antibodies used in flow cytometry.

Antibody	Fluorochrome
CD45	AF700
CD3	PerCP
CD8	APCH7
CD4	PerCpy5
CD38	APC
HLA-DR	V500
CD45RA	PEcy7
CCR7	PE
CCR6	BV605
CXCR3	AF800

The strategy used for the analysis of the different lymphocytes subpopulations is provided in **Supplementary Figure S1**.

### 2.5 Indirect basophil activation test

For the indirect BAT, seven healthy volunteers aged between 21 and 38 years old were selected. These volunteers had the 0+ blood group to avoid nonspecific reactions involving ABO antibodies in the patients’ serum, and they were designated as basophil donors throughout the time of the study.

Protocol used is based on several studies involving patients diagnosed with CSU (22–24). Briefly, 50 µl of patient’s serum was incubated with 50 µl of basophil donor’s blood. After incubation for 15 min at 37°C, samples were lysed and washed with reagents provided by the kit. Basophils were identified using the cell surface expression of CCR3 in the Side Scatter Chanel (SSC) and activated basophils according CD63 expression. Cells were acquired and analyzed using CCR3 expression acquiring a minimum of 500 total basophils in a FACSLyrics cytometer (BD Biosciences). The strategy used in the analysis is provided in **Supplementary Figure S2**. Antibodies anti-FcεRI and N-formyl-methionyl-leucyl-phenylalanine (fMLP) were used as positive controls.

It was considered positive when (1) the percentage of CD63+ basophils is >5% and (2) stimulation index (SI) is ≥2 [SI = percentage of CD63+ cells induced by the patients’ serum divided by the percentage of CD63+ cells induced by the negative control] (22–24).

### 2.6 Statistical analysis

The statistical analysis of the population was performed using univariate analysis to determine the relationship between each variable. This was achieved using the χ² for qualitative variables and the student *t*-test for quantitative variables. For non-parametric data, *U* Mann–Whitney test was used.

Subsequently, a multivariate analysis was performed with logistic regression. All independent variables which in the univariate analysis showed a relationship with the main variable with a *P*-value of < 0.5 were included in the model, considering that *p < 0.05,**p < 0.01, ***p < 0.001.

Statistical analysis was performed using the GNU PSPP program version 1.2.0-g0fb4db and GraphPad Prism 7.03.

## 3 Results

### 3.1 Patients’ characterization

#### 3.1.1 Clinical characteristics

Of the 33 patients receiving omalizumab treatment, 26 patients (77.7%) were women. Eighteen patients (57%) were enrolled from the Allergology Section and 14 patients (43%) from Dermatology Department. Angioedema occurred in nine patients (30%).

Antithyroid antibodies were found in five patients (16%). Nine patients had concomitant autoimmune disease (ulcerative colitis, autoimmune thyroiditis, type 1 diabetes, vitiligo, or psoriasis). The majority were women (seven of nine), with four experiencing angioedema and two having atopy. One patient had a dose reduction, and one did not respond to the treatment.

More than half of the patients (52.63%) had positive skin tests to inhalant allergens (mostly to dust mites) and 18.2% had positive specific IgE to anisakis, but no positivity to other hidden allergens such as Pru p3 or Tri a 19.

#### 3.1.2 UAS 7 score and response to omalizumab

All patients started omalizumab and 26 patients (79%) completed the treatment course. Seven patients (21%) discontinued treatment due to lack of efficacy.

The mean UAS 7 score before omalizumab treatment was 30.1 (from 28 to 42: severe). When comparing the UAS 7 score results before (UAS 7 = 30) and after omalizumab treatment (UAS 7 = 5), a notable reduction in scores was observed (*p* < 0.0001), indicating a significant enhancement in the patient’s quality of life. Additionally, the cohort was stratified into those who did not complete the treatment due to inefficacy and those who complete the full dose. Changes in the UAS 7 score after initiating omalizumab remained statistically significant (*p* < 0.05), with the group that completed the treatment showing a lower UAS7 score (**Supplementary Table S1**).

#### 3.1.3 Inflammation markers and serum proteins

CSU patients had significantly elevated serum IgE levels despite omalizumab treatment (mean IgE in omalizumab patients: 362.87 ± 902.5 kU/L; mean IgE in NID patients: 157 ± 306.3 kU/L; normal range <100 kU/L).

In our study, there were no differences in others serum markers, except for C-reactive protein (CRP), where higher levels were seen in patients treated with omalizumab. (**Supplementary Table S2**).

### 3.2 Immune profile

#### 3.2.1 Indirect BAT test is not affected by omalizumab treatment

Initially, the BAT technique was validated using 10 healthy controls (being all of them negative).

Subsequently, sera from the various patient groups were analysed (*n* = 45), with only 34% yielding positive BAT result. Among the positive cases, only eight patients were undergoing omalizumab treatment. No correlation could be identified between BAT positivity and anti-IgE treatment.

#### 3.2.2 Patients receiving omalizumab treatment exhibited a distinct profile in T-cell subsets compared to those undergoing treatment with NIDs

In the analysis of T lymphocyte subpopulations and phenotype characterization, we analyzed 24 patients who had been undergoing omalizumab treatment for 6 months and 27 patients under NIDs. Both groups were then compared with a control group of 50 healthy individuals. All the changes in the percentages and absolute counts of different T lymphocyte subpopulations between groups are provided in **Supplementary Tables S3**, **S4**.

**Figure 2:**
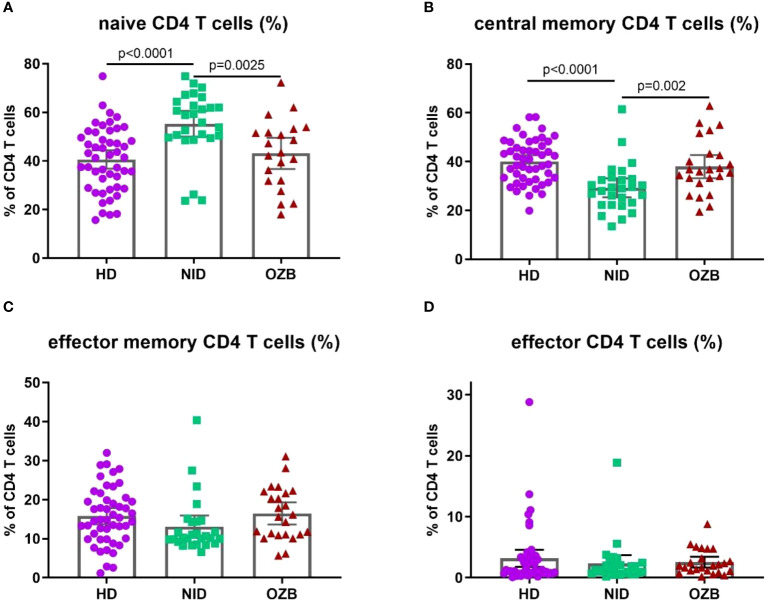
Differences in the percentages of naïve and central memory CD4 T cells were observed in patients with CSU treated with omalizumab when compared to those treated with NID or to HD. Percentages of **(A)** naïve, **(B)** central memory, **(C)** effector memory, and **(D)** effector CD4 T cells in peripheral blood of HD, NID, and CSU patients under omalizumab treatment. CSU, chronic spontaneous urticaria; HD, healthy donors (*n* = 50); NID, non-immunomodulatory drugs (*n* = 27); OZB, omalizumab (*n* = 24).

In relation to CD4^+^ T lymphocytes, patients with CSU under NID treatment had higher percentages of naïve cells and lower percentages of central memory subsets than HD (naïve CD4: 58.90 [23.6–74.9] vs. 42 [15.7–74.9] %, *p* = 0.0001, and central memory CD4: 29.10 [13.4–61.5] vs. 39.95 [2.1–58.3] %, *p* = 0.0001; **Figure 2**). In contrast, in treatment with omalizumab there were lower percentages of naïve cells and higher of central memory cells,when compared to NID, restoring values similar to those found in HD (naïve CD4: 43.40 [22.1–62] vs. 58.90 [23.6–71.9] %, *p* = 0.0025, and central memory CD4: 36.35 [19.5–62.9] vs. 29.10 [13.4–61.5] %, *p* = 0.0020; **Figure 2**).

**Figure 3:**
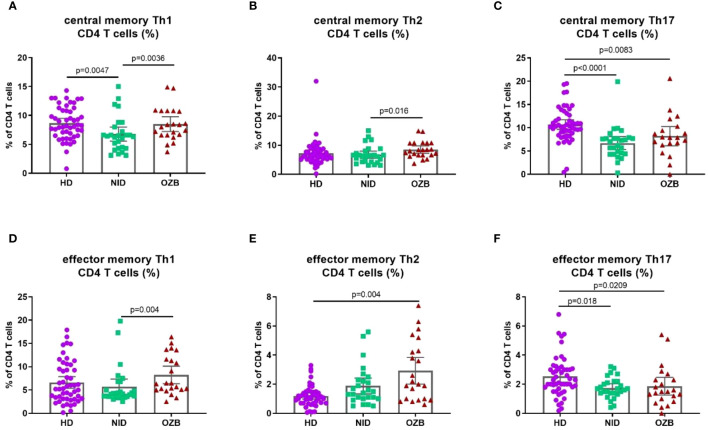
Th1 and Th2, but not Th17; CD4 T subsets are affected by treatment with omalizumab. Percentages of **(A)** central memory Th1 **(B)** central memory Th2, **(C)** central memory Th17, **(D)** effector memory Th1, **(E)** effector memory Th2, and **(F)** effector memory Th17 T cells in peripheral blood of HD, NID, and CSU patients under omalizumab treatment. CSU, chronic spontaneous urticaria; HD, healthy donors (*n* = 50); NID, non-immunomodulatory drugs (*n* = 27); OZB, omalizumab (*n* = 24).

Regarding functional T subsets (**Figure 3**), in CSU, there was a decrease of the percentage of Th1 cells when compared with HD, which was reverted in the treatment with omalizumab. On the other hand, no differences were observed between HD and NID patients concerning Th2 cells, but, interestingly, their percentages were increased in the treatment with omalizumab. In contrast, percentages of Th17 cells were decreased in CSU patients with NID but omalizumab had not effect on them.

**Figure 4:**
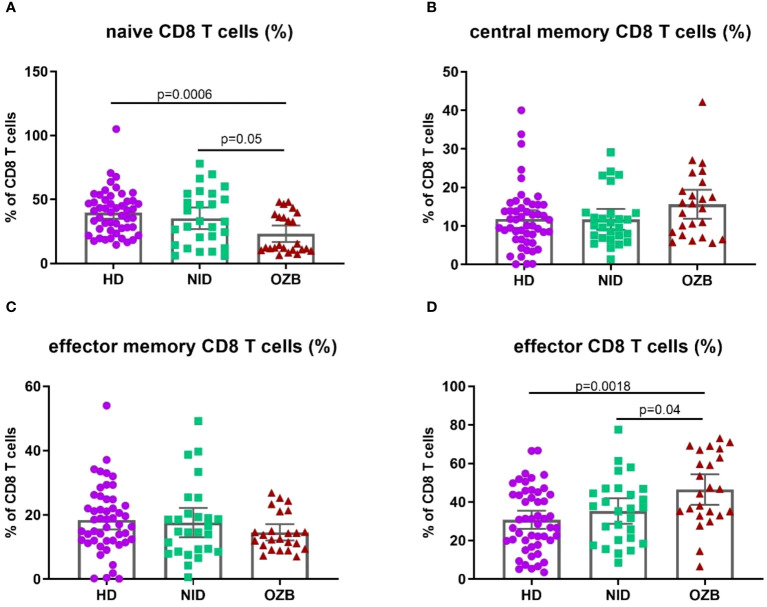
Differences in the percentages of CD8 T-cell subpopulations in patients under treatment with omalizumab compared to HD and treated with NID. Percentages of **(A)** naïve, **(B)** central memory, **(C)** effector memory, and **(D)** effector CD8 T cells in peripheral blood of HD, NID and CSU patients under omalizumab treatment. CSU, chronic spontaneous urticaria; HD, healthy donors (*n* = 50); NID, non-immunomodulatory drugs (*n* = 27); OZB, omalizumab (*n* = 24).

In CD8^+^ T cells, patients under treatment with omalizumab had lower percentages of naïve cells and higher percentages of the effector subsets than NID patients and HD (naïve CD8: 13.40 [6.5–48.2] vs. 38.75 [10.5–70.7] %, *p* = 0.0002. and effector CD8: 45.60 [6.6–73.1] vs. (29.70 [3.5–66.70] %, *p* = 0.0007; **Figure 4**).

**Figure 5:**
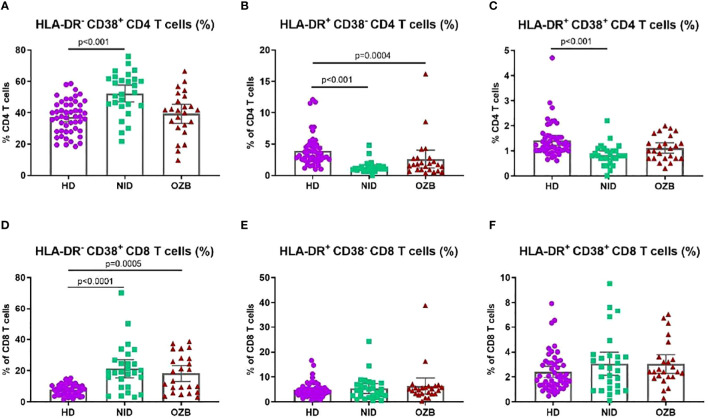
Changes in the percentage of CD4 and CD8 T cells expressing CD38 and HLA-DR in peripheral blood of CSU treated with NID, treated with omalizumab and HD. Percentages of **(A)** HLA-DR^-^CD38^+^ CD4 T cells, **(B)** HLA-DR^+^CD38^-^ CD4 T cells, **(C)** HLA-DR^+^CD38^+^ CD4 T cells, **(D)** HLA-DR^-^CD38^+^ CD8 T cells, **(E)** FILA-DR^+^CD38^-^ CD8 T cells, **(F)** HLA-DR^+^CD38^+^ CD8 T cells in peripheral blood of HD, NID and CSU patients under omalizumab treatment. CSU, chronic spontaneous urticaria; HD, healthy donors (*n* = 50); NID, non-immunomodulatory drugs (*n* = 27); OZB, omalizumab (*n* = 24).

When we analyzed activation markers, we found that the percentages of CD4^+^ and CD8^+^ T cells expressing CD38 but not HLA-DR were increased in CSU patients treated with NID and not affected in omalizumab treatment. Moreover, CD4^+^ T cells expressing HLA-DR were lower in peripheral blood of patients with CSU (**Figure 5**).

Moreover, we analyzed if there was a correlation between inflammatory markers, presence of autoantibodies and indirect BAT positivity with T-cell subsets. No correlation was found (data not shown).

#### 3.2.3 Omalizumab induces a decrease in naïve CD8^+^ T cells and an increase of Th1 and Th2 subsets

**Figure 6:**
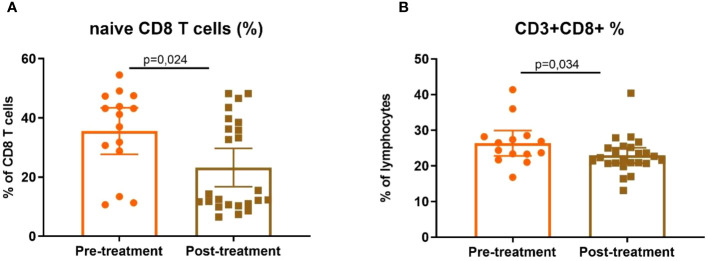
Differences in CD8 subpopulations before and after treatment. Percentages of **(A)** CD8 naïve (CD3^+^CD8^+^CD27^+^CCR7^+^CD45RA^+^) and **(B)** total CD8 T cells in peripheral blood in patients before (*n* = 14) and 6 months after treatment (*n* = 24) with omalizumab.

To confirm the correlation between the impact of anti-IgE treatment and potential alterations in lymphocyte subpopulations, T lymphocyte subpopulations were analysed in CSU patients undergoing omalizumab before starting and 6 months after treatment with it (*n* = 38, 14 baseline patients and 24 post-treatment). Prior to treatment, patients displayed elevated levels of naïve CD8^+^ T cells (41.2 [10.7–54.5] vs. 13.4 [6.5–48.2] %, *p* = 0.024) and total CD8^+^ T cells (25.45 [16.8–41.4] vs. 22.15 [13.1–40.4] %, *p* = 0.034) (**Figure 6**).

Regarding CD4^+^ T-cell subsets, although statistically significant differences were not observed, there was a notable trend towards an increase in activated CD4^+^ HLA-DR^+^CD38^+^ T cells and the subpopulations of Th1 effector memory and Th2 central memory in patients once they are under treatment with omalizumab (**Supplementary Figure S3**).

Finally, we studied lymphocyte subpopulations in a subgroup of nine CSU patients before and after treatment. Although no significant differences were identified, likely attributed to the small sample size, a tendency toward a decrease in CD4^+^HLA-DR^+^CD38^-^T cells and an increase in central and effector Th1 memory subpopulations, as well as activated CD4^+^ HLA-DR^+^CD38^+^ T cells, was observed during the treatment with omalizumab (**Supplementary Figure S4**).

## 4 Discussion

The main goal of our study was to investigate cellular immunity of patients with CSU and its relationship with the response to omalizumab treatment.

Managing and monitoring patients with CSU is challenging. Therefore, treatment with a monoclonal antibody as omalizumab is essential to prevent specific IgE from binding to FcεRI on mast cells and enhance the patient’s quality of life. Most of our patients responded positively to treatment although in 40% of them, a dose increase was necessary. These results were also reflected in the UAS 7 score, where scores significantly decreased after omalizumab treatment, confirming the improvement of patients once treatment was initiated (1, 25).

Regarding immune response and the impact of omalizumab on the immune system, several studies have indicated a correlation between indirect BAT results and the response to anti-IgE treatment (16, 26, 27). The indirect BAT has established itself as a specific and sensitive *in-vitro* test to identify autoantibodies IgG in patients with CAU (6). Several studies have investigated the usefulness of utilizing BAT as a tool for diagnosis and monitoring patients with CSU, yet it remains a matter of controversy (28). In our cohort, only eight patients had positive indirect BAT results, of which three were under omalizumab treatment. In the present study, indirect BAT has demonstrated to be useless; and should not be introduced in the clinical practice.

Although omalizumab is an effective treatment for CSU patients, its mechanism of action remains elusive. Effects on mast cell activation, downregulation of IgE receptors, reversal basophenia or reducing IgG autoantibodies against IgE or its receptor, have been described but are not yet fully understood (25). Furthermore, the potential influence on T cells remains unknown. Studies in cohorts with other allergic diseases (such as asthma) could be useful to assess the effect of omalizumab on adaptive immune responses.

Regarding the involvement of T cells in the pathogenesis of CSU, it has been described that, in skin biopsies of CSU patients, there is a perivascular non-necrotizing cellular infiltration with predominance of CD4 T cells, not only of Th2 but also of Th1 and Th17 subsets (29). Moreover, these CD4 T cells found in the skin expressed high levels of HLA-DR (12). Here, we described that patients with CSU had in peripheral blood low percentages of not only CD4 T cells expressing HLA-DR but also Th1, Th2, and Th17 cells. These results support that these CD4 T cells migrate to the skin where they participate in the pathogenesis of the wheal. Also, we found an increase in the percentage of naïve T cells and a decrease of memory subsets in the peripheral blood of patients with CSU. Although, as far as we know, this is the first study analyzing naïve and memory T-cell subsets in patients with CSU, these results also support an infiltration of memory T cells in the skin lesions.

Interestingly, treatment with omalizumab induced changes in naïve and memory subsets of T cells, as well as in Th1 and Th2 subsets, in most of the cases restoring values like those found in the HD group. It has been described that omalizumab decreases the expression of FcεRI on dendritic cells (30). Moreover, it is known that dendritic cells are key players in the differentiation of Th1 and Th2 subsets. Although more studies should be performed to better understand the mechanism of action of omalizumab in those T-cell subsets, an effect of omalizumab in dendritic cells could explain why under the treatment the percentages of Th1 and Th2 subsets are restored to levels like HD.

However, the analysis of activation markers and Th17 cells could be used to characterize patients with CSU but are not modified by treatment with omalizumab, so they are not of interest for our goal of monitoring of the treatment.

Despite the findings, our study has limitations too. It is a preliminary study with a small cohort of CSU patients treated with omalizumab, and these findings should be validated in a larger cohort. Additionally, although we described changes in T-cell subpopulations induced by omalizumab, the mechanism of how the drug modifies these T-cell phenotypes is still not fully clear. Further studies should be assessed in the future to better characterize it.

In summary, in this paper, we postulate that changes in the T lymphocyte subpopulations can be used as putative markers of the treatment effect. Hence, during the follow-up of patients starting treatment with omalizumab, we can expect a decrease in the percentage of naïve and increase of central memory CD4^+^ T cells, a decrease in naïve and increase of effector CD8^+^ T-cell subsets, as well as an increase of Th1 and Th2 cells but the mechanism of how omalizumab modifies T-cell phenotype is not fully understood. Further studies are needed to elucidate its mechanism of action in T cells.

## 5 Conclusion

The analysis by flow cytometry of T-cell subsets in peripheral blood of CSU patients undergoing treatment with omalizumab would be useful in the clinical practice to better characterize the effect of the treatment in each patient.

## Data Availability

The original contributions presented in the study are included in the article/**Supplementary Material**. Further inquiries can be directed to the corresponding authors.
