# Supplementary figures and images for: Peripheral blood T-cell modulation by omalizumab in chronic urticaria patients
Source: Front Immunol. 2024 Aug 20;15:1413233. doi: 10.3389/fimmu.2024.1413233 (PMC11368771; doi:10.3389/fimmu.2024.1413233)

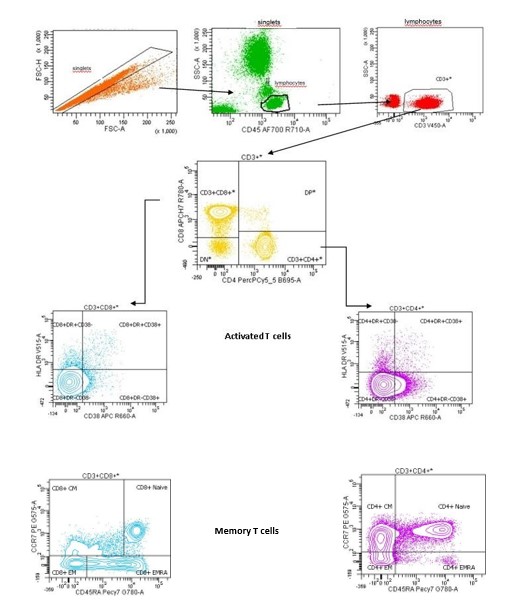

Supplement: Supplementary file 1 [file Image1.jpeg]

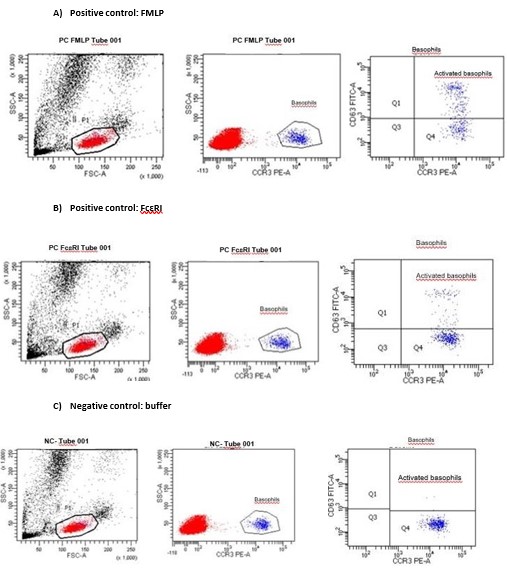

Supplement: Supplementary file 2 [file Image2.jpeg]
