# Supplementary material for: Peripheral blood T-cell modulation by omalizumab in chronic urticaria patients
Source: Front Immunol. 2024 Aug 20;15:1413233. doi: 10.3389/fimmu.2024.1413233 (PMC11368771; doi:10.3389/fimmu.2024.1413233)
Supplement: Supplementary file 3 [file Image3.pdf]

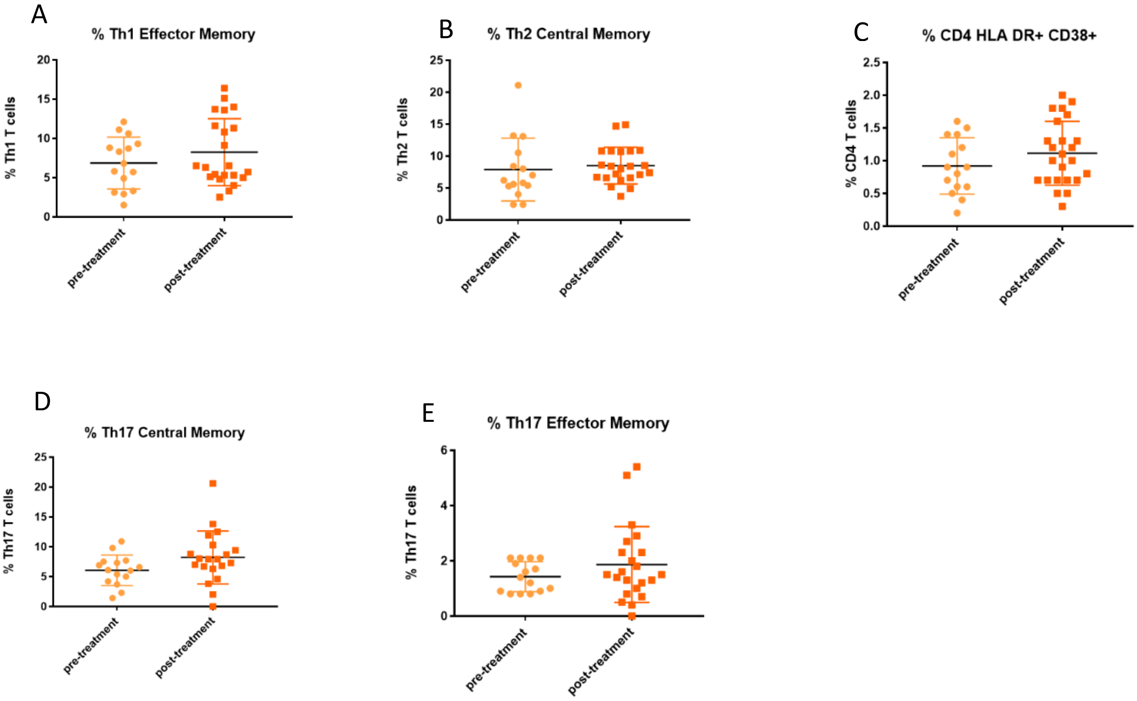

Figure S3: Differences in T helper subsets before and after treatment with omalizumab. Percentages of (A) Th1 Effector Memory ($CD3^{+}CD4^{+}CCR7^{+}CD45RA^{-}CXCR3^{+}CCR6^{-}$), (B) Th2 Central Memory ($CD3^{+}CD4^{+}CCR7^{+}CD45RA^{-}CXCR3^{-}CCR6^{-}$), (C) activated CD4 ($CD3^{+}CD4^{+}HLA\text{-}DR^{+}CD38^{+}$), (D) Th 17 Central Memory ($D3^{+}CD4^{+}CCR7^{+}CD45RA^{-}CXCR3^{-}CCR6^{+}$), (E) Th 17 Effector Memory ($CD3^{+}CD4^{+}CCR7^{+}CD45RA^{-}CXCR3^{-}CCR6^{+}$) T cells in peripheral blood of patients before(n=14) and 6 months after(n=24) treatment with omalizumab.
