# Supplementary material for: Peripheral blood T-cell modulation by omalizumab in chronic urticaria patients
Source: Front Immunol. 2024 Aug 20;15:1413233. doi: 10.3389/fimmu.2024.1413233 (PMC11368771; doi:10.3389/fimmu.2024.1413233)
Supplement: Supplementary file 4 [file Image4.pdf]

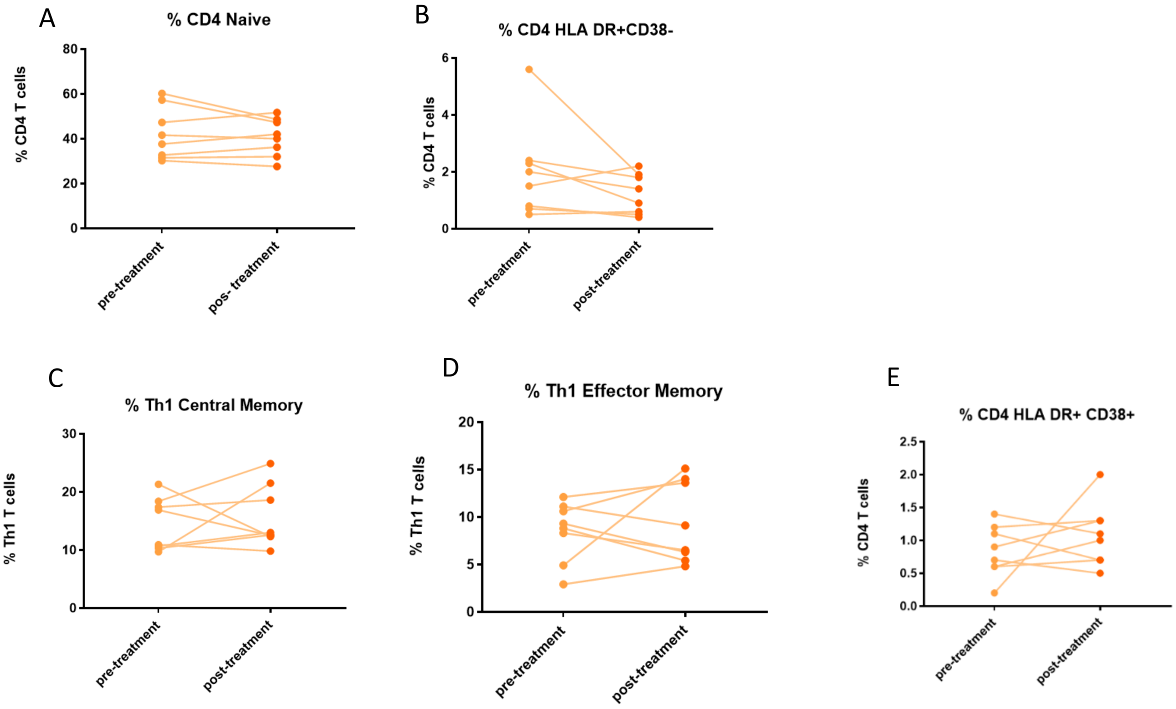

Figure S4. Differences in CSU patients before and after treatment with omalizumab (N=9). Percentages of (A) CD4 naïve (($CD3^+CD4^+CD27^+CCR7^+CD45RA^+$), (B) activated CD4 ($CD3^+CD4^+HLA\text{-}DR^+CD38^-$), (C) Th1 Central Memory ($CD3^+CD4^+CCR7^-CD45RA^-CXCR3^+CCR6^-$), (D) Th1 Effector Memory ($CD3^+CD4^+CCR7^+CD45RA^-CXCR3^+CCR6^-$),(E) activated CD4 ($CD3^+CD4^+HLA\text{-}DR^+CD38^+$) T cells in peripheral blood in patients before and 6 months after treatment with omalizumab.
