# Supplementary material for: Peripheral blood T-cell modulation by omalizumab in chronic urticaria patients
Source: Front Immunol. 2024 Aug 20;15:1413233. doi: 10.3389/fimmu.2024.1413233 (PMC11368771; doi:10.3389/fimmu.2024.1413233)
Supplement: Supplementary file 5 [file Table1.docx]

| Variables | Omalizumab group  (N=33) |
| --- | --- |
| Age, mean ±SD, years | 48,19±14,43 |
| Female gender, N (%) | 26 (77.7) |
| Associated autoimmune disease, N (%) | 6 (17.3) |
| Associated angioedema, N(%) | 10 (30.3) |
| Associated thyroid Antibodies, N(%) | 5 (16) |
| Positive skin prick test to inhalants, N(%) | 17 (52.6) |
| Positive IgE anisakis, N (%) | 6 (18.2) |
| UAS-7 Score, median | 30.1 |
| Erythrocyte Sedimentation rate (ml/h), median | 16.50 |
| C- Reactive Protein (mg/l), median | 25.50 |
| D-dimer (μg/ml), median | 107 |
