# Supplementary material for: Peripheral blood T-cell modulation by omalizumab in chronic urticaria patients
Source: Front Immunol. 2024 Aug 20;15:1413233. doi: 10.3389/fimmu.2024.1413233 (PMC11368771; doi:10.3389/fimmu.2024.1413233)
Supplement: Supplementary file 6 [file Table2.docx]

| Variables  (median, IQ) | CSU/Pre-Omalizumab  (N=23) | CSU/Post-Omalizumab  (N=11) | CSU/Non-Omalizumab  (N=27) | P  Pre vs post omalizumab | P  Post vs Non-Omalizumab | P  Pre vs Non-omalizumab |
| --- | --- | --- | --- | --- | --- | --- |
| Age, years | 48±14,43 | 48±14,43 | 41.8±20,7 | ns | ns | ns |
| Female, gender, n (%) | 26 (77.7) | 26 (77.7) | 19 (67.8) | ns | ns | ns |
| D-Dimer, μg/mL | 326(27- 1402) | 107(2.02-477) | 310 (63-1326) | 0.072 | **0.023** | 0.80 |
| Total IgE, IU/mL | 185 (2-1895) | 402 (9.6-1922) | 111 (29.1-1411) | 0.117 | 0.024 | 0.276 |
| ESR, ml/h | 13 (2-65) | 16.50(2-25) | 8.50(2-36) | 0.321 | 0.058 | 0.471 |
| CRP, mg/L | 4.6 (0.1-35.1) | 25.50 (0.3-21) | 1.6 (0.3-35.1) | **0.034** | **0.0001** | **0.0369** |
| BAT, positive, N | x | 8 | 7 |  |  |  |
