# Supplementary material for: Peripheral blood T-cell modulation by omalizumab in chronic urticaria patients
Source: Front Immunol. 2024 Aug 20;15:1413233. doi: 10.3389/fimmu.2024.1413233 (PMC11368771; doi:10.3389/fimmu.2024.1413233)
Supplement: Supplementary file 7 [file Table3.docx]

| Subpopulations | Parent | Percentage (%)  Healthy donors  (median, IQ) | | Percentage  (%) NID  (median, IQ) | | Percentage (%)  Omalizumab  (median, IQ) | | HD VS NID  **(p)** | HD Vs OZB  **(p)** | NID VS OZB  **(p)** |
| --- | --- | --- | --- | --- | --- | --- | --- | --- | --- | --- |
| **CD4^+^ T lymphocytes** | T cells | **62.28** | [44.4-83.9] | **42.7** | [25.1-69.9] | **47.9** | [32.9-63] | **<0.0001** | **<0.001** | 0.38 |
| **CD4 T naïve** | T CD4 | **42** | [15.7-74.9] | **58.90** | [23.6-74.9] | **43.40** | [18.0-72.3] | **<0.0001** | 0.628 | **0.0025** |
| **CD4 T central memory** | T CD4 | **39.95** | [19.9-58.3] | **29.10** | [13.4-61.5] | **36.35** | [19.5-62.9] | **<0.0001** | 0.362 | **0.0020** |
| **CD4 T effector memory** | T CD4 | **14.85** | [1.2-32] | **10.30** | [6.6-40.4] | **15.90** | [5.7-28.1] | **0.0457** | 0.65 | **0.012** |
| **CD4 T EMRA** | T CD4 | **1.46** | [0.1-28.8] | **1.3** | [0.5-18.9] | **2.0** | [0.2-8.8] | 0.356 | 0.644 | 0.186 |
| **CD4 T DR+CD38+** | T CD4 | **1.23** | [0.65-4.71] | **0.80** | [0.2-2.2] | **1.05** | [0.3-1.8] | **<0.001** | 0.058 | 0.053 |
| **CD4 T DR+CD38-** | T CD4 | **3.07** | [0.96-12.1] | **1.1** | [0.6-4.8] | **1.7** | [0.5-16.2] | **0.0001** | **<0.0001** | 0.073 |
| **CD4 T DR-CD38+** | T CD4 | **37.7** | [18.4-58.6] | **53** | [21.7-71.4] | **41.60** | [15.6-66.6] | **<0.0001** | 0.41 | **0.0013** |
| **Th1 CM** | T CD4 CM | **8.6** | [5.03-14.30] | **6.4** | [3.1-12.9] | **8.2** | [3.7-14.9] | **0.0026** | 0.695 | **0.017** |
| **Th1 EM** | T CD4 EM | **5.25** | [0.15-17.9] | **4.2** | [2.5-19.8] | **6.4** | [2.5-16.4] | 0.485 | 0.084 | **0.004** |
| **Th2 CM** | T CD4 CM | **6.55** | [0.22-13.8] | **6.4** | [3.1-15.0] | **8.2** | [3.7-14.9] | 0.57 | **0.022** | **0.0168** |
| **Th2 EM** | T CD4 EM | **1.125** | [0.13-10.8] | **1.50** | [0.5-20.5] | **2.10** | [0.6-7.4] | **0.034** | **0.0025** | 0.224 |
| **Th17 CM** | T CD4 CM | **10.28** | [0.46-19.5] | **7.0** | [0.3-19.9] | **7.90** | [0.0-20.6] | **<0.0001** | **0.0033** | 0.115 |
| **Th17 EM** | T CD4 EM | **2.3** | [0.18-20.12] | **1.7** | [0.4-3.1] | **1.5** | [0.0-5.1] | **0.036** | **0.0135** | 0.671 |
| **CD8^+^ T lymphocytes** | T cells | **31.3** | [12.7-48.5] | **23.5** | [13.2-35.4] | **23.6** | [16.4-40.4] | **<0.0001** | **0.0005** | 0.09 |
| **CD8 T naïve** | T CD8 | **38.75** | [10.5-70.7] | **32.8** | [5.9-78.1] | **13.4** | [6.5-48.2] | 0.519 | **0.0002** | 0.057 |
| **CD8 T central memory** | T CD8 | **10.85** | [0.1-33.8] | **10.1** | [1.4-29.1] | **15.20** | [5.6-42.2] | 0.81 | 0.059 | 0.090 |
| **CD8 T effector memory** | T CD8 | **17.75** | [0.1-54] | **14.80** | [0.6-49.2] | **13.20** | [7-26.9] | 0.412 | 0.073 | 0.56 |
| **CD8 T EMRA** | T CD8 | **29.7** | [3.5-66.7] | **36.6** | [8.5-77.6] | **45.6** | [14.6-73.1] | 0.33 | **0.0007** | 0.042 |
| **CD8 T DR+CD38+** | T CD8 | **2.00** | [0.48-6.52] | **2.7** | [0.1-9.5] | **2.4** | [0.3-6.7] | 0.37 | 0.077 | 0.862 |
| **CD8 T DR+CD38-** | T CD8 | **4.57** | [0.76-11.2] | **4.7** | [0.5-24.2] | **5.05** | [0.3-38.9] | 0.97 | 0.486 | 0.719 |
| **CD8 T DR-CD38+** | T CD8 | **7.87** | [1.52-14.6] | **19.10** | [3-70.2] | **16.45** | [3-38.6] | **<0.0001** | **0.0004** | 0.782 |
