# Supplementary material for: Peripheral blood T-cell modulation by omalizumab in chronic urticaria patients
Source: Front Immunol. 2024 Aug 20;15:1413233. doi: 10.3389/fimmu.2024.1413233 (PMC11368771; doi:10.3389/fimmu.2024.1413233)
Supplement: Supplementary file 8 [file Table4.docx]

| Subpopulations | Parent | Absolute count (cel/μl)  Healthy donors  (median,IQ) | | Absolute count(cel/μl)  NID  (median,IQ) | | Absolute count(cel/μl)  Omalizumab  (median,IQ) | | HD VS NID  **(p)** | HD Vs OZB  **(p)** | NID VS OZB  **(p)** |
| --- | --- | --- | --- | --- | --- | --- | --- | --- | --- | --- |
| **CD4^+^ T lymphocytes** | T cells | **807.5** | [323-1520] | **1022** | [549-2167] | **964** | [526-2079] | **0.0097** | 0.085 | 0.386 |
| **CD4 T naïve** | T CD4 | **307.5** | [92-682] | **572** | [137-1623] | **401.5** | [167-727] | **0.0004** | 0.254 | **0.028** |
| **CD4 T central memory** | T CD4 | **293.5** | [13-813] | **278** | [130-459] | **315.5** | [179-840] | 0.179 | 0.57 | 0.113 |
| **CD4 T effector memory** | T CD4 | **130** | [7-336] | **109.5** | [57-406] | **143** | [59-300] | 0.617 | 0.1545 | 0.102 |
| **CD4 T EMRA** | T CD4 | **12** | [1-170] | **13** | [1-128] | **18.50** | [1-63] | 0.83 | 0.704 | 0.583 |
| **CD4 T DR+CD38+** | T CD4 | **12.5** | [4-32] | **6.5** | [2-28] | **7.5** | [2-27] | **0.0002** | **0.004** | 0.294 |
| **CD4 T DR+CD38-** | T CD4 | **26** | [7-155] | **11** | [4-48] | **17** | [3-206] | **<0.0001** | **0.0101** | 0.161 |
| **CD4 T DR-CD38+** | T CD4 | **279.5** | [120-776] | **497.5** | [204-1149] | **370** | [114-869] | **0.0007** | 0.199 | **0.036** |
| **Th1 CM** | T CD4 CM | **67** | [5-198] | **84** | [31-328] | **121** | [46-302] | 0.221 | **0.022** | 0.134 |
| **Th1 EM** | T CD4 EM | **47** | [1-176] | **42** | [15-201] | **55** | [27-221] | 0.899 | 0.091 | 0.076 |
| **Th2 CM** | T CD4 CM | **50** | [1-148] | **49** | [28-179] | **72** | [40-227] | 0.592 | 0.0548 | 0.124 |
| **Th2 EM** | T CD4 EM | **10** | [1-66] | **13** | [3-115] | **21** | [4-94] | **0.037** | **0.0019** | 0.374 |
| **Th17 CM** | T CD4 CM | **83** | [3-219] | **56.5** | [4-197] | **70** | [20-150] | **0.0044** | 0.1912 | 0.296 |
| **Th17 EM** | T CD4 EM | **17.5** | [2-168] | **16** | [3-42] | **13.50** | [4-60] | 0.251 | 0.28 | 0.972 |
| **CD8^+^ T lymphocytes** | T cells | **351.5** | [134-1011] | **502** | [236-1218] | **471** | [238-646] | **0.0045** | 0.314 | 0.095 |
| **CD8 T naïve** | T CD8 | **135.5** | [31-543] | **145.5** | [28-503] | **68.50** | [27-311] | 0.915 | **0.008** | 0.084 |
| **CD8 T central memory** | T CD8 | **43** | [1-143] | **57.5** | [10-119] | **64** | [13-259] | 0.121 | **0.040** | 0.378 |
| **CD8 T effector memory** | T CD8 | **71.5** | [1-229] | **77** | [4-248] | **63.5** | [21-140] | 0.59 | 0.41 | 0.31 |
| **CD8 T EMRA** | T CD8 | **133** | [11-427] | **181.5** | [40-446] | **179.5** | [43-339] | 0.090 | **0.040** | 0.835 |
| **CD8 T DR+CD38+** | T CD8 | **10.5** | [1-489 | **14.5** | [3-63] | **11** | [1-27] | 0.75 | 0.590 | 0.374 |
| **CD8 T DR+CD38-** | T CD8 | **17** | [1-86] | **28** | [1-121 | **17.5** | [1-182] | 0.30 | 0.5 | 0.617 |
| **CD8 T DR-CD38+** | T CD8 | **24.5** | [5-119] | **87** | [11-350] | **54.5** | [22-158] | **0.0001** | **0.0038** | 0.177 |
